# NDM-1 Introduction in Portugal through a ST11 KL105 *Klebsiella pneumoniae* Widespread in Europe

**DOI:** 10.3390/antibiotics11010092

**Published:** 2022-01-12

**Authors:** Ângela Novais, Rita Veiga Ferraz, Mariana Viana, Paula Martins da Costa, Luísa Peixe

**Affiliations:** 1 UCIBIO, Laboratório de Microbiologia, Departamento de Ciências Biológicas, Faculdade de Farmácia, Universidade do Porto, 4050-313 Porto, Portugal; 2 Associate Laboratory i4HB—Institute for Health and Bioeconomy, Faculty of Pharmacy, University of Porto, 4050-313 Porto, Portugal; 3 Centro Hospitalar do Tâmega e Sousa, 4564-007 Penafiel, Portugal; rita.ferraz@chts.min-saude.pt (R.V.F.); 60565@chts.min-saude.pt (M.V.); pcmcosta@live.com.pt (P.M.d.C.); 4 Department of Clinical Chemistry, Centro Hospitalar Universitário do Porto, 4099-001 Porto, Portugal

**Keywords:** nosocomial infections, carbapenemase-producing Enterobacterales, NDM-1, whole-genome sequencing, outbreak, Fourier transform infrared spectroscopy, infection control

## Abstract

The changing epidemiology of carbapenem-resistant *Klebsiella pneumoniae* in Southern European countries is challenging for infection control, and it is critical to identify and track new genetic entities (genes, carbapenemases, clones) quickly and with high precision. We aimed to characterize the strain responsible for the first recognized outbreak by an NDM-1-producing *K. pneumoniae* in Portugal, and to elucidate its diffusion in an international context. NDM-1-producing multidrug-resistant *K. pneumoniae* isolates from hospitalized patients (2018–2019) were characterized using FTIR spectroscopy, molecular typing, whole-genome sequencing, and comparative genomics with available *K. pneumoniae* ST11 KL105 genomes. FT-IR spectroscopy allowed the rapid (ca. 4 h after incubation) identification of the outbreak strains as ST11 KL105, supporting outbreak control. Epidemiological information supports a community source but without linkage to endemic regions of NDM-1 producers. Whole-genome comparison with previous DHA-1-producing ST11 KL105 strains revealed the presence of different plasmid types and antibiotic resistance traits, suggesting the entry of a new strain. In fact, this ST11 KL105 clade has successfully disseminated in Europe with variable beta-lactamases, but essentially as ESBL or DHA-1 producers. We expand the distribution map of NDM-1-producing *K. pneumoniae* in Europe, at the expense of a successfully established ST11 KL105 *K. pneumoniae* clade circulating with variable plasmid backgrounds and beta-lactamases. Our work further supports the use of FT-IR as an asset to support quick infection control.

## 1. Introduction

The dissemination of carbapenem resistant *K. pneumoniae* (CRKP) is a well-known problem, but there is a great asymmetry in the geographic distribution of carbapenemase types and *K. pneumoniae* lineages across countries [1,2,3]. Moreover, changing epidemiology over time is a very challenging situation that affects several countries from Southern Europe [4,5,6,7]. Knowing and to understanding these dynamics is essential in order to promptly recognize new genetic entities with enhanced antibiotic resistance, virulence and/or transmission, and to allow a prompt redesign of diagnostic tools and infection control policies to curtail further dissemination [2,8]. Whole-genome sequencing has provided the greatest resolution in establishing transmission pathways at local and global levels of CRKP [1,2], and in enlightening the phylogeny and evolutionary history of some highly frequent KP clonal groups (e.g., CG258, CG307) [8,9,10]. However, it is still unable to support real-time infection control decisions.

ST11 is one of the most diffused clones, and is especially prevalent in Asia and particularly in China [11]. The heterogeneity within ST11 is well recognized in studies on this geographic area, with clades reflecting capsule recombination events (KL64, KL47, KL105) [9,12]. For this reason, because Fourier transform infrared (FT-IR) spectroscopy is a suitable tool to identify *K. pneumoniae* capsular (KL) types with a fast turnaround time [13,14], it is ideal to discriminate and identify ST11 variants, which are understudied in Europe.

In Portugal, resistance to carbapenems is a problem with growing proportions, especially among *K. pneumoniae*, of which the rates among invasive isolates recorded in national surveillance networks increased ca. 80% in just three years (2014–2017) [15]. Reporting of CPE from national laboratories has been mandatory since 2013 and there have been official regulations for the management of outbreaks and for CPE infection control since 2017 [4]. The available epidemiological and population analysis data suggest the dominance all over the country of particular genetic traits (the ST147 clone, KPC-3 carbapenemase) [16,17,18,19], with an increasing diversity of species, carbapenemases types and clones [2,4,20]. KPC-3 and to a lesser extent OXA-48 or OXA-181, which are occasionally associated with GES-5 [18,20], are the most common carbapenemase types among *K. pneumoniae*. NDM-1 has been sporadically described in *Providencia stuartii*, *Morganella morganii* and *Proteus mirabilis* [21,22] but, prior to this study, no cases of colonization and/or infection with NDM-producing *K. pneumoniae* were reported. NDM-1 is especially prevalent in India, the Middle East and the Balkans, and its emergence in other countries (especially in *Escherichia coli* or *K. pneumoniae*) is considered a public health priority due to the risk of subsequent (frequently polyclonal) dissemination [3,23,24].

In this study, we used FT-IR spectroscopy to support the identification and control of the first recognized outbreak of NDM-1-producing ST11 capsular-type KL105 *K. pneumoniae* in Portugal. Furthermore, we used whole-genome sequencing to trace the variability and diffusion of this particular ST11 *K. pneumoniae* clade from an international perspective.

## 2. Results

### 2.1. Epidemiological Context and Infection Control Measures

We identified and characterized the first recognized outbreak of NDM-1-producing *K. pneumoniae* isolates in Portugal, involving a total of seven patients. The index case was from a patient (patient 1) with no history of previous hospitalization or travel abroad, whereas most other cases were linked to concomitant hospitalization in the surgical unit at the same time as the index patient (patients 2–6) (Table 1). None of the other patients had a record of foreign travel in the 3 months prior to strain isolation. Patient 3 had been previously hospitalized in a different hospital but screening at admission was negative. Patient 7 came from a household and no epidemiological link could be established with other patients, but this patient had also been previously hospitalized and tested negative at first admission.

The patient cohort, screening of putative carriers, contact precautions and the reinforcement of cleaning and disinfection practices prevented new cases. Regular screening of CPE carriers at admission and during hospitalization, as well as weekly audits to assess compliance to the protocols and implement corrective measures, were used to monitor for the appearance of new cases, and these are still in practice to control the spread of CPE.

In the three months prior to NDM-1 detection, the patients who received antibiotherapy were treated with beta-lactam/beta-lactamase inhibitors, cefuroxime, ceftazidime and ciprofloxacin.

### 2.2. Strain Relatedness

FT-IR spectroscopy was performed retrospectively on suspected isolates received at the Faculty of Pharmacy, University of Porto. After reception, our FT-IR based workflow allowed us to establish a close relationship between the first five isolates, and to communicate strain relatedness less than 4 h after bacterial culture. Comparison of the spectra with our database allowed the classification of the K-type as KL105, together with previous DHA-1 producing ST11-KL105 isolates that had been circulating in Portuguese hospitals for years (Figure S1) [25]. The four isolates identified subsequently clustered with these, confirming the K-type and strain relatedness by means of FTIR, which was further confirmed using PFGE for all studied isolates (data not shown). The genes *bla*_NDM-1_, as well as *bla*_CTX-M-15_, were detected in all isolates via PCR, and all isolates exhibited identical susceptibility profiles.

### 2.3. Molecular Characterization

Isolates revealed multidrug resistance profiles, being resistant to all antibiotics tested except amikacin, tigecycline and colistin. Sequenced isolates carried several antibiotic resistance genes (*bla*_NDM-1_, *bla*_SHV-182_, *bla*_CTX-M-15_, *aac(6′)-Ib-cr*, *aac(3)-IId*, *aph(3′’)-Ib*, *aph(6)-Id*, *oqxAB*, *qnrB1*, *catB3*, *sul2*, *tetD* and *dfrA14*), supporting the phenotypic data. They also carried chromosomal mutations in *gyrA* (S83I) and *parC* (S80I), and UhpT (E350Q), conferring reduced susceptibility to fluoroquinolones and fosfomycin, respectively. They were also enriched with virulence genes encoding enterobactin (*entB*), yersiniabactin (*irp1/2*, *ybt10* associated with ICEKp4), iron receptors (*fyuA*, *kfu*BC) and type I and type III fimbriae (*fimH*, *mrk*ABC). All strains had *wzi*75, which corresponds to a novel capsular type (KL105), and KLEBORATE confirmed 100% identity with the KL105 locus and the O2v2 O antigen locus.

### 2.4. Whole-Genome Data and Phylogenetic Analysis

**Figure 1:**
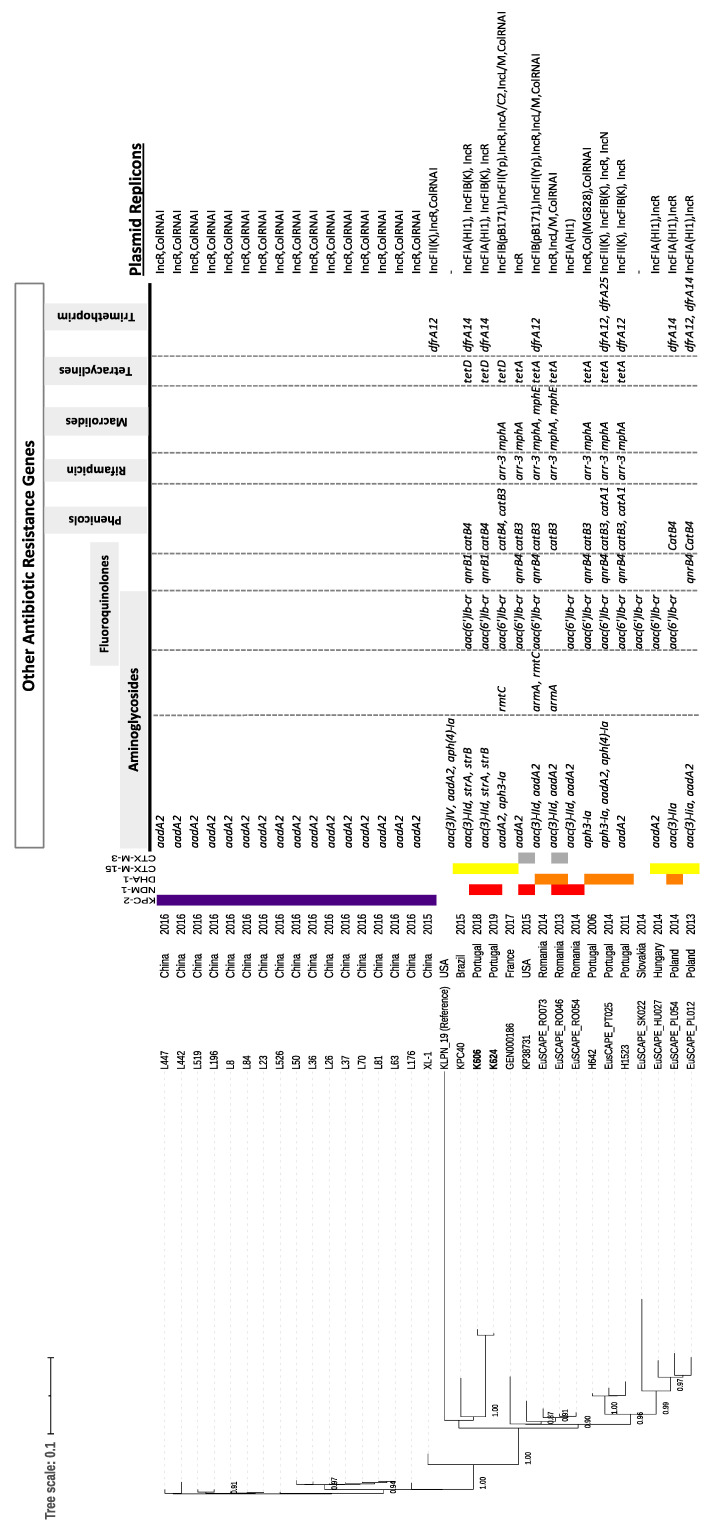
SNP-based phylogenetic tree of ST11-KL105 genomes and heatmap of antimicrobial resistance genotype. Genomes sequenced from this study (shown in bold) were compared with those from 28 other ST11-KL105 strains deposited in public databases (accessed on 5 November 2019). Phylogenetic tree was obtained using the SNP method of CSI phylogeny with default parameters and the reference strain KLPN_19, the closest ST11 with KL107 (accession number GCF_003861305.1). Country and year of isolation are shown, as represented in metadata, as well as their content of acquired beta-lactamases (ESBL, acquired AmpC and carbapenemases), other antibiotic resistance genes and replicons. Numbers in the left indicate bootstrap values >0.85, calculated based on the maximum-likelihood method.

The genomes of K606 and K624 strains had 5.5 Gb, a GC content of 57.2% and were assembled into 76–97 contigs (data not shown). The SNP-based phylogenetic tree was constructed on the basis of 4.87 Gb common positions covered by the core genome, corresponding to 88.5% of the genome of the reference strain. We observed a close relatedness between the available ST11 KL105 genomes from Europe, America and China (2013–2017) since isolates varied at 0–297 sites distributed over their 4.8 million base pair (bp) core genome (~medium 31SNP/Gb) (Figure 1, Supplementary Figure S2). All of them carried *bla*_SHV-182_ in the chromosome.

Two main branches were identified within the tree (Figure 1). One of these included highly related (<20 SNPs) KPC-2-producing strains from China and carried IncR and ColRNAI plasmids and a few additional antibiotic resistance genes. The second branch included isolates producing CTX-M-3, CTX-M-15 or DHA-1, occasionally together with NDM-1, most of them (n = 13/15; 87%) identified in Europe, and many from the Euscape project [2]. Most (92%) of these carried several antibiotic resistance genes, some of which were signatures of particular genetic platforms (e.g., *catB3*, *arr-3*, *qnrB4*). Most carried R replicons, but also FIA(HI1), FIIk and/or FIB, together with variable F replicons (FIA, FIB and/or FIIk, and colRNAI) (Figure 1). All of them consistently carried *ybt10* (ICEKp4), *ipr1/irp2* and *fyuA2* virulence genes.

The SNP-based matrix (supplementary Figure S2) showed that the two sequenced strains differ in only six SNPs, supporting their close relatedness. However, due to the absence of an epidemiological link between both patients, it is reasonable to consider an unrecognized source. Both strains showed ~100 SNPs (20 SNP/Gb) with previous DHA-1 ST11-KL105 producers circulating in Portuguese hospitals (Whole Genome Shotgun project accession Nº QTTC00000000 and QTTD00000000). Three of the public genomes, corresponding to isolates from Romania and the USA, also carried *bla*_NDM-1_ and showed only 80–90 SNP differences with our outbreak strains.

### 2.5. Genetic Support for bla_NDM-1_ Acquisition

**Figure 2:**
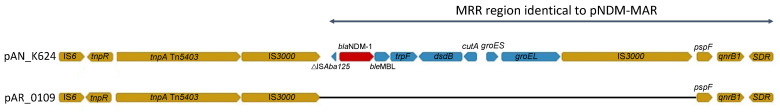
Genetic environment of *bla*_NDM-1_ in plasmid pAN_K624, represented using Geneious version 9.1.8. The location of the insertion of the MRR region into the pAR_0109-like plasmid backbone is shown.

The *bla*_NDM-1_ gene was identified in pAN_K624, an 84 Kb IncR+IncFIA(HI1) multirreplicon plasmid with high identity (100%) and coverage (92%) to the pAR_0109 plasmid (Genbank acc. Number CP032212), which is of an unknown origin and which carries the typical genetic environment of *bla*_CTX-M-15_. *bla*_NDM-1_ is located in a Tn*125*-like multidrug resistance region (MRR) comprising deltaIS*Aba125*, *bla*_NDM-1_, *ble*_MBL_, *trpF*, *dsbC*, *cutA*, *groES*, *groEL*, *psp*, *qnrB1* and SDR family reductase, 99% identical to that of the reference plasmid pNDM-MAR (Genbank acc. Number JN420336). The MRR region (~8.5Kb) containing *bla*_NDM-1_ was flanked by an IS*3000* element that might have mediated its insertion into the backbone of a pAR_0109-like plasmid between a Tn*5403*-like transposon and *pspF* (Figure 2).

Additionally, these strains also carried a 149 Kb IncFIB(K) plasmid with high identity and coverage (>99%) with the ~160 Kb p002SK2_A plasmid (Genbank acc. Number CP025516), a non-antibiotic resistant plasmid identified in a K. pneumoniae ST147 isolate from wastewater in Switzerland. There were no traces of the DHA-1-producing pKPS30 IncR plasmid previously identified in ST11-KL105 isolates circulating in Portugal [25], nor of the IncC plasmid previously detected with bla_NDM-1_ in species other than K. pneumoniae [21]. Likewise, none of the other NDM-1-producing ST11 KL105 public strains carried a similar plasmid replicon content, suggesting a new plasmid as the vehicle of bla_NDM-1_.

## 3. Discussion

This study enlarges the distribution map of NDM-1 carbapenemase in Europe and raises the alarm for its emergence in our country in a multidrug-resistant ST11-KL105 *K. pneumoniae* lineage, that has previously caused the long-term dissemination of DHA-1 in Portuguese hospitals [2,25]. Simultaneously, it increases the pool of carbapenemases circulating among high-risk *K. pneumoniae* lineages in our country, which is worrisome considering the possibility of further transmission of *bla*_NDM-1_ to other already circulating high-risk *K. pneumoniae* clones or even to other Enterobacterales [4,17]. In fact, other NDM-1-producing *K. pneumoniae* ST11-KL105 strains with identical PFGE patterns have been more recently identified in other hospitals in our area, suggesting further dissemination and wider expansion (data not shown).

It is of interest to highlight that none of the patients had a record of hospitalization or travel abroad in the 3 months prior to strain isolation, and the absence of an epidemiological link for the index case suggests community acquisition from an unrecognized source. A chain of nosocomial dissemination could be established, and our data further reinforce the importance of contact screening of CPE using fast and reliable methods such as FT-IR, together with quick identification of carbapenemases, in order to optimize infection control, to detect new emergences and prevent subsequent spread [4].

The quick identification of the outbreak using FT-IR spectroscopy (ca. 4 h after incubation) allowed the prompt establishment of infection control measures for outbreak control, and whole-genome sequencing supported the classification of the outbreak strain as ST11 KL105. Our comparative genomic analysis of available ST11 KL105 genomes revealed that this lineage has been found circulating in several European countries since at least 2006, in most cases carrying *bla*_DHA-1_, *bla*_CTX-M-3_ or *bla*_CTX-M-15_ (Figure 1). This is in agreement with data from the Euscape project [2], in which ca. 62% of ST11 *K. pneumoniae* isolates were non-carbapenemase producers. Here, we additionally demonstrate that these Euscape isolates belong to the same ST11 clade expressing KL105, which differs from variants prevalent in China and eastern European countries [9,12,26].

Our comparative genomic analysis shows that NDM-1 was not acquired by ST11 KL105 strains previously circulating in Portugal [25] but through the introduction of a new ST11 KL105 strain, carrying *bla*_NDM-1_ in a different genetic context. In fact, comparison of NDM-1- and DHA-1-producing ST11 strains revealed variability of beta-lactamases (*bla*_NDM-1_, *bla*_CTX-M-15_ vs *bla*_DHA-1_), antibiotic resistance traits (*qnrB1*, *catB3*, *dfrA14* vs *qnrB4*, *arr-3*, *catA1*, *dfrA12*) and plasmids (IncR+FIA(HI1) + IncFIB(K) vs IncR). The pAN_K624 plasmid (IncR+FIA(HI1)) encoding NDM-1 was also different from the IncC platform identified previously in other Enterobacterales in Portugal [21], supporting a new entry of the *bla*_NDM-1_ gene and the circulation of different platforms encoding NDM-1. Furthermore, our data on the plasmid genetic context suggest the introduction of *bla*_NDM-1_ into a multidrug resistance plasmid that is different from those carrying *bla*_NDM-1_ in other ST11 KL105 strains from Europe, suggesting a new platform. Also, it differs from the pKPX-1 plasmid, associated with massive NDM-1 spread in Poland [26]. In the ST11 KL105 clade, the acquisition of *bla*_NDM-1_ seems to be sporadic, in some cases over DHA-1 or ESBL genetic backgrounds (Figure 1, Supplementary Table S1), contributing to high genetic plasticity and plasmid shuffling in this lineage, extending previous observations [27].

Our work also highlights the need to further deepen the population analysis of *K. pneumoniae* clones to obtain maximum discrimination for the efficient tracking of outbreak strains. Indeed, there is high genetic variability within some of the most frequent clonal groups, including CG11 [2,27,28]. Despite the high capsular diversity identified among the most common clonal groups, many studies have demonstrated an extraordinary selection of lineages with specific capsular types within CG258 [28] and CG11 [9,12], suggesting that the capsule type is a good evolutionary marker of clinically relevant *K. pneumoniae* lineages [13]. Considering this evolutionary scenario, FT-IR based typing based on a representative spectral database is a reliable tool to differentiate these lineages, providing information that, together with epidemiological data, might be of great value in supporting the identification of outbreaks and real-time infection control decisions at a quick and low-cost rate [13,14,29]. Whole-genome sequencing requires a higher cost and time but it can be used downstream to provide full resolution, allowing a thorough understanding of the dynamics of the spread of antimicrobial resistance [8,10,27].

This ST11-KL105 *K. pneumoniae* lineage consistently carries *bla*_SHV-182_, encoding a natural SHV-type beta-lactamase, according to the BLBD beta-lactamase database (http://bldb.eu accessed on 11 November 2019) [30]. In all the genomes analyzed, *bla*_SHV-182_ was chromosomally located and might be potentially used as a marker for this specific lineage.

## 4. Materials and Methods

### 4.1. Setting

The outbreak occurred at a 500-bed hospital that serves a population of about 520,000 in the north of Portugal, together with another hospital unit at a distance of 30 km. It offers healthcare specialties within medical, surgical and emergency (adults and pediatric), as well as outpatient attendance. Control of the dissemination of CPE bacteria is implemented according to official national recommendations and includes active screening and the reinforcement of infection control procedures. At this hospital, rectal screening of CPE is performed (i) at admission when transferring from another hospital with hospitalizations >48 h or from a household or long-term care facility; (ii) when there are previous (12 months) hospitalizations, (iii) every 7 days of hospitalization. At admission, genes encoding specific carbapenemases were detected using a rapid PCR kit (Xpert CarbaR), whereas subsequent screenings on hospitalized patients were performed using a cultural method.

### 4.2. Index Case

The first NDM-1-producing *K. pneumoniae* was isolated in the surgical ward in the bile of a 71-year-old patient with a cholangiocarcinoma on 27 November 2018. In the absence of clinical symptoms it was considered possible drain colonization, and CPE rectal screening performed the day after revealed the carriage of a NDM-1-producing isolate. This patient had been hospitalized since 26 October 2018 but there were no criteria for screening at admission, thus this information is missing.

These isolates were resistant to third-generation cephalosporins, carbapenems, aztreonam, beta-lactam/beta-lactamase inhibitors, fluoroquinolones, trimethoprim-sulfamethoxazole, nitrofurantoin and several aminoglycosides; intermediate to fosfomycin; and susceptible only to amikacin, tigecycline and colistin.

### 4.3. Additional Cases

Six additional patients were identified with NDM-1-encoding *K. pneumoniae*. All patients in the same nursery were considered exposed and were screened for rectal carriage independently on the result at admission (if applicable). Three of them carried a *K. pneumoniae* isolate with the same susceptibility profile. All patients with positive screening results were transferred to cohort areas and subjected to isolation and contact precautions. Four additional isolates were subsequently identified in the rectal swabs or caused a urinary infection in three outpatients that were admitted between December 2018 and March 2019 from the emergency room. Two of them were identified as carriers of NDM-producing *K. pneumoniae* at admission, whereas the last one was negative for carriage at admission during a previous hospitalization but that missed subsequent screenings.

### 4.4. Bacterial Identification and Carbapenemase Detection

In total, 9 NDM-1-encoding *K. pneumoniae* isolates were identified in seven patients. These isolates were recovered from feces (n = 9), urine (n = 1) and bile (n = 1) (Table 1). Carbapenemase genes were primarily detected via molecular biology using Xpert CarbaR (Cepheid). Bacterial identification and preliminary susceptibility testing were performed using a VITEK 2 system (bioMérieux) or broth microdilution (for colistin). Available clinical and epidemiological data, as well as information on recognized risk factors for infection by CPE, were recorded and analyzed.

### 4.5. Antibiotic Resistance Phenotypes and Genotypes

Antibiotic susceptibility profiles to 25 antibiotics (all beta-lactams, beta-lactam/beta-lactamase combinations, fluoroquinolones, aminoglycosides, tetracyclines, fosfomycin, trimethoprim-sulfamethoxazole, chloramphenicol) were determined via disk diffusion and interpreted following EUCAST guidelines (www.eucast.org 6 January 2020). Carbapenemase production was confirmed using the Blue-Carba test [31] and the type of carbapenemase was identified via PCR directed to the most frequent carbapenemase gene families (*bla*_KPC_, *bla*_OXA-48_, *bla*_IMP_; *bla*_VIM_, *bla*_NDM_) and further sequencing [17,32]. The presence of *bla*_CTX-M-15_ was confirmed using PCR in all isolates.

### 4.6. Identification of Outbreak Strains

We used Fourier transform infrared (FTIR) spectroscopy to assess isolates’ relationships according to our previously established workflow and models based on the comparison of bacterial discriminatory spectra. The analysis was performed retrospectively on suspected isolates received in early December 2018 and March 2019. Upon reception, isolates were re-grown in standardized culture conditions (37 °C/18 h), after which a colony was directly deposited on the ATR accessory of our FT-IR instrument (Perkin-Elmer Frontier) and air-dried. Three spectra per strain were acquired (technical replicates) from 600 cm^−1^ to 4000 cm^−1^, and the spectral region of interest (accumulating polysaccharide vibrations) was compared with spectra included in our in-house *K. pneumoniae* database and machine-learning model [13]. The K-type and clone of the suspected strains were then predicted with an accuracy of 100%, because of previously established correlations in internationally circulating lineages [13]. These FT-IR-based assignments were confirmed by means of PCR and sequencing of the *wzi* gene [33], as well as pulsed-field gel electrophoresis.

### 4.7. Whole Genome and Phylogenetic Analysis

Two NDM-1-producing isolates (the first and the last outbreak strain with no epidemiological link) were selected for whole genome sequencing. The DNA was extracted using the Wizard Genomic DNA purification kit (Promega Corporation, Madison, WI, USA) according to the manufacturer’s instructions and the concentration was determined with a Qubit 3.0 Fluorometer (Invitrogen, Thermo Fisher Scientific, Waltham, MA, USA). DNA was sequenced using an Illumina Novaseq 6000 (2× 300 bp pair-ended runs, ~6 Gb genome, coverage 100×), reads were trimmed de novo using Trimommatic 0.39 to remove adapters (http://www.usadellab.org/cms/?page=trimmomatic accessed on 5 November 2019) and assembled using SPAdes version 3.9.0 (cab.spbu.ru/software/spades/ accessed on 5 November 2019). The quality of the reads and the assembly were assessed using FastQC v0.11.9 (http://www.bioinformatics.babraham.ac.uk/projects/fastqc/ accessed on 5 November 2019) and Quast (http://cab.cc.spbu.ru/quast/ accessed on 5 November 2019). Contigs were further annotated with RAST (https://rast.nmpdr.org accessed on 6 November 2019). This Whole Genome Shotgun project has been deposited at DDBJ/ENA/GenBank under the BioProject accession number PRJNA484888, with Biosamples SAMN16522901 and SAMN16522902.

Provided with a snapshot of the ST11 clade distribution in previous papers [9,12], we decided to focus on understanding the phylogeny and geographic distribution of the ST11 KL105 clade since these isolates were poorly represented in those series. Twenty-eight ST11-KL105 *K. pneumoniae* genomes available on GenBank database (https://www.ncbi.nlm.nih.gov/genbank accessed on 18 November 2019) were included for comparison (Supplementary Table S1). Antibiotic resistance and replicon content were extracted from Center for Genomic Epidemiology tools (ResFinder, PlasmidFinder at http://www.genomicepidemiology.org accessed on 5 November 2019) or CARD (https://card.mcmaster.ca accessed on 5 November 2019). Plasmid contigs were identified using mlplasmids [34]. Plasmid assembly and partial reconstruction was obtained, mapping ST11 contigs on reference plasmid sequences and BLAST (https://blast.ncbi.nlm.nih.gov/Blast.cgi accessed on 5 November 2019). Geneious version 9.1.8 was used to represent the *bla*_NDM-1_ genetic environment.

The virulence gene content and *wzi*, capsular (KL) and O antigen loci were assessed using the *K. pneumoniae* Institut Pasteur database (https://bigsdb.pasteur.fr/cgi-bin/bigsdb/bigsdb.pl?db=pubmlst_klebsiella_seqdef accessed on 5 November 2019) and KLEBORATE (https://github.com/katholt/Kleborate accessed on 5 November 2019). The core genome of the two sequenced isolates (K606 and K624), the two previously described DHA-1-producing ST11-KL105 strains from Portugal [25] and those of 26 other ST11-KL105 strains deposited in the GenBank database were extracted and used to construct a SNP-based phylogenetic tree using the CSI phylogeny tool, using default parameters (https://cge.cbs.dtu.dk/services/CSIPhylogeny/ accessed on 6 November 2019) and the maximum-likelihood method to calculate bootstrap values. The closest genome corresponding to strain KLPN_19 (a ST11 strain with KL107 GCF_003861305.1) was used as a reference, and the tree was further represented with Interactive Tree of Life (iTOL, https://itol.embl.de accessed on 7 November 2019).

## 5. Conclusions

Data from the first recognized outbreak of NDM-1-producing *K. pneumoniae* in our country raise the alarm for the potential silent dissemination of NDM-1 through unnoticed carriage in the community, which needs to be monitored to prevent further spread. This report extends the distribution map of NDM-1 carbapenemase in Europe, which emerged through the re-introduction of ST11 KL105 *K. pneumoniae* clade into our country and a new plasmid backbone carrying *bla*_NDM-1_. We provide the first comparative analysis of the ST11 KL105 *K. pneumoniae* clade and show that it is successfully established in European countries carrying a variable pool of ESBL, DHA-1 or NDM-1-encoding plasmids. Finally, we further demonstrate the usefulness of the speed and the accuracy of FT-IR spectroscopy to support outbreak identification and infection control.

## Figures and Tables

**Table 1:** Epidemiological and clinical data of patients colonized/infected by NDM-1-producing *K. pneumoniae* ST11-KL105.

Patient N°	Sex/Age	Isolates	Date of Isolation	Hospital Unit	Period of Hospitalization	Patient’s Origin	Sample Type	Pathology	Colonization at Admission	Antibiotherapy (Previous 3 Months)	Previous Hospitalization (Date, Unit)
1	M/71	K607 K606 *	27/11/18 28/11/18	Surgery	26–30 October 2018	Hospital	C (bile) C (RS)	Cholangiocarcinoma	Not tested	Piperacillin-Tazobactam	None
2	M/79	K608	28/11/18	Surgery	15 November–3 December 2018	Hospital	C (RS)	Acute pancreatitis	Not tested	None	None
3	M/55	K609	30/11/18	Surgery	24 October–5 December 2018	Hospital	C (RS)	Trauma	-	Amoxacillin-clavulanic acid	15 September–22 October 2018 (different hospital)
4	M/66	K610	30/11/18	Surgery	23–30 November 2018	Hospital	C (RS)	Adenocarcinoma	Not tested	None	None
5	M/66	K611	14/12/19	Emergency	14 December 2018–24 January 2019	Outpatient	C (RS)	Colon carcinoma	+	Ciprofloxacin	10–28 November 2018 (Surgery)
6	M/68	K612	25/12/19	Emergency	25 December 2018–11 January 2019 (Medicine)	Outpatient	C (RS)	Soft tissue infection	+	Tigecycline, Ceftazidime	31 October–9 December 2018 (Surgery)
7	M/87	K624 * K625	25/03/19	Emergency	25 March–18 April 2019	Household	I (urine) C (RS)	Pneumonia	-	Cefuroxime	28 January–4 March 2019

* Isolates selected for whole-genome sequencing. M = male; C = colonization; I = infection; RS = rectal swab.

## Data Availability

This Whole Genome Shotgun project has been deposited at DDBJ/ENA/GenBank under the BioProject accession number PRJNA484888, with Biosamples SAMN16522901 and SAMN16522902.

## Supplementary Materials

The following are available https://www.mdpi.com/article/10.3390/antibiotics11010092/s1, Table S1: List of *K. pneumoniae* ST11-KL105 genomes deposited on NCBI public databases used for phylogenetic analysis. Figure S1: Projection of FT-IR spectra from the outbreak *K. pneumoniae* isolates (gray dots) in the in-house partial-least squares discriminant model (PLSDA) [13]. Figure S2: SNP matrix obtained from core-genome DNA comparison between *K. pneumoniae* ST11 KL105 genomes, supporting phylogenetic tree.
