# Supplementary material for: NDM-1 Introduction in Portugal through a ST11 KL105 Klebsiella pneumoniae Widespread in Europe
Source: Antibiotics (Basel). 2022 Jan 12;11(1):92. doi: 10.3390/antibiotics11010092 (PMC8773016; doi:10.3390/antibiotics11010092)
Supplement: Supplementary file 1 [file antibiotics-11-00092-s001.zip › antibiotics-1494526-supplementary.pdf]

Supplementary Materials

NDM-1 Introduction in Portugal through a ST11 KL105 *Klebsiella pneumoniae* Widespread in Europe

Ângela Novais [1,2,*], Rita Veiga Ferraz [3], Mariana Viana [3], Paula Martins da Costa [3,4] and Luísa Peixe [1,2,*]

1 UCIBIO. Laboratório de Microbiologia, Departamento de Ciências Biológicas, Faculdade de Farmácia, Universidade do Porto, 4050-313 Porto, Portugal
2 Associate Laboratory i4HB—Institute for Health and Bioeconomy, Faculty of Pharmacy, University of Porto, 4050-313 Porto, Portugal
3 Centro Hospitalar do Tâmega e Sousa, Penafiel, Portugal; rita.ferraz@chts.min-saude.pt (R.V.F.); 60565@chts.min-saude.pt (M.V.); pcmcosta@live.com.pt (P.M.d.C.)
4 Centro Hospitalar Universitário do Porto, Porto, Portugal
* Correspondence: aamorim@ff.up.pt; (Â.N.); lpeixe@ff.up.pt (L.P.); Tel.: +351-220-428-580 (Â.N. or L.P.).

Table S1. List of *Klebsiella pneumoniae* ST11-KL105 genomes deposited on NCBI public databases used for phylogenetic analysis.

Strain Name	Accession number	Country	Isolation Date
L176	GCF_002262735.1	China	2016
L37	GCF_002262415.1	China	2016
L36	GCF_002262385.1	China	2016
L50	GCF_002262115.1	China	2016
L26	GCF_002262455.1	China	2016
L519	GCF_002261945.1	China	2016
L447	GCF_002262145.1	China	2016
L23	GCF_002262565.1	China	2016
L196	GCF_002262635.1	China	2016
L84	GCF_002261575.1	China	2016
L526	GCF_002261935.1	China	2016
L8	GCF_002261685.1	China	2016
L442	GCF_002262175.1	China	2016
L70	GCF_002261655.1	China	2016
L81	GCF_002261615.1	China	2016
L63	GCF_002261785.1	China	2016
XL-1	GCF_001939845.1	China	2015
KPC40	GCF_002209465.1	Brazil	2015
K606	SAMN16522901	Portugal	2018
K624	SAMN16522902	Portugal	2019
KP38731	GCF_002009075.1	USA	2015
EuSCAPE_RO073	GCF_900504255.1	Romania	2014
EuSCAPE_RO046	GCF_900504005.1	Romania	2013
EuSCAPE_RO054	GCF_900504085.1	Romania	2014
GEN000186	GCF_004146185.1	France	2017
EuSCAPE_PT025	GCF_900502825.1	Portugal	2014
H1523	GCA_009746735.1	Portugal	2011
H642	GCA_009746705.1	Portugal	2006
EuSCAPE_SK022	GCF_900510755.1	Slovakia	2014
EuSCAPE_HU027	GCF_900516865.1	Hungary	2014
EuSCAPE_PL054	GCF_900510385.1	Poland	2014
EuSCAPE_PL012	GCF_900511375.1	Poland	2013

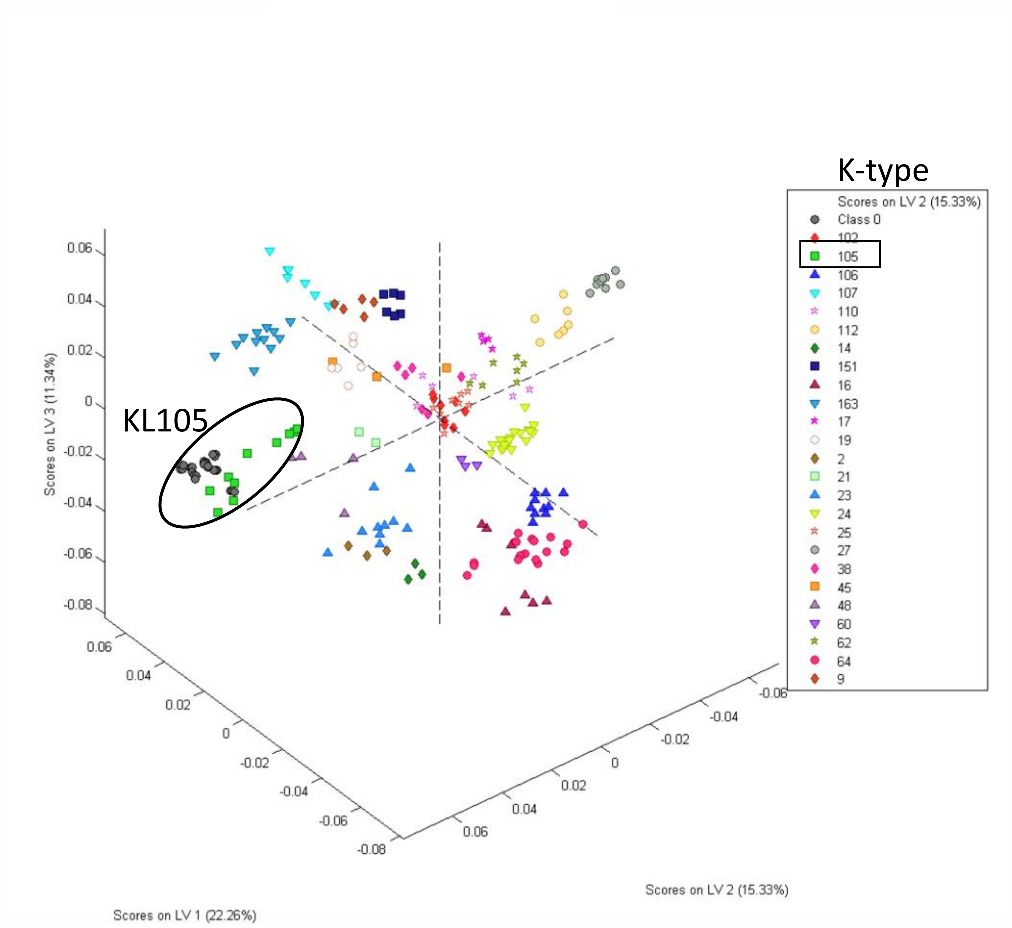

Figure S1. Projection of FT-IR spectra from outbreak *K. pneumoniae* isolates (grey dots) in the in-house partial-least squares discriminant model (PLSDA) discriminating 25 K-types.

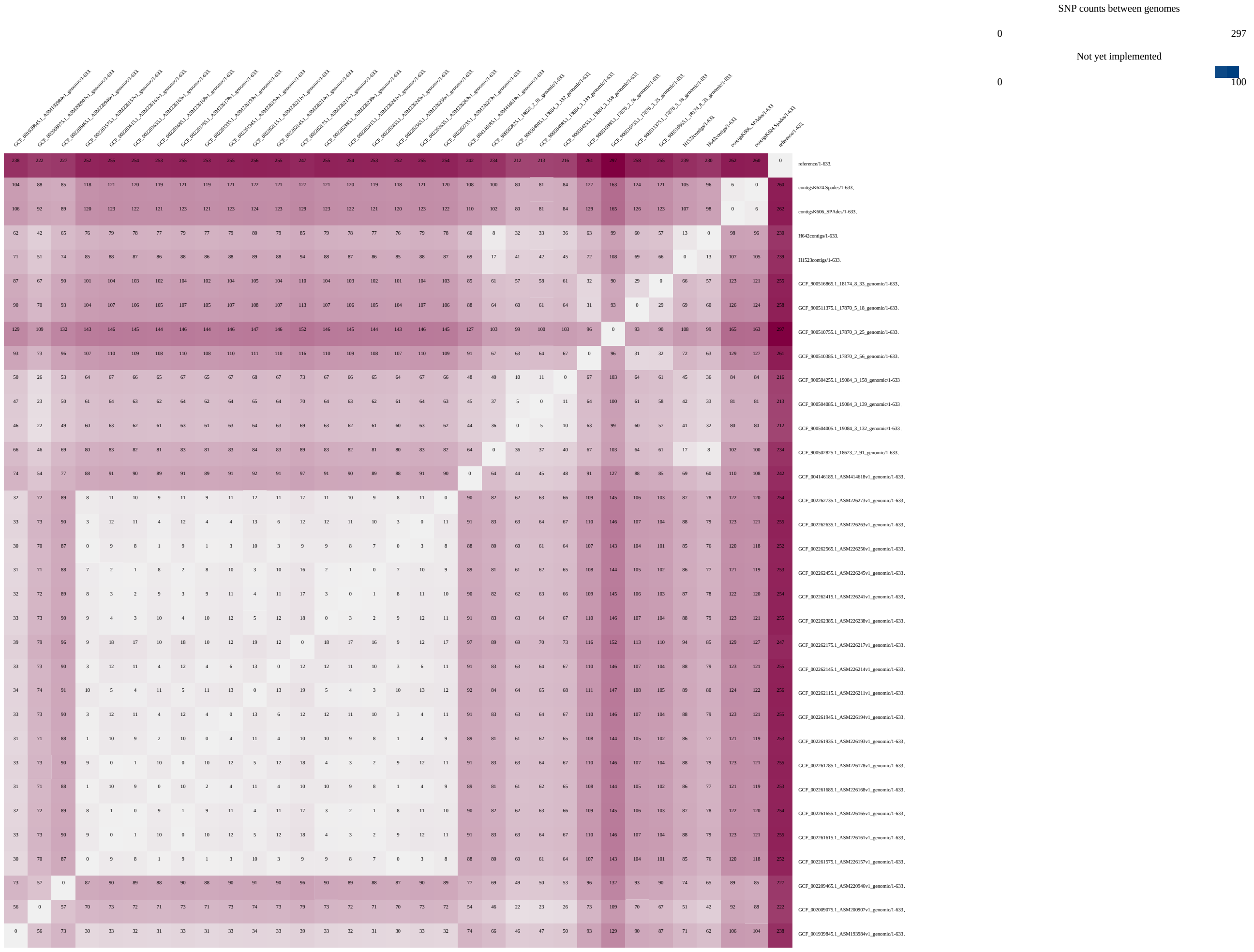

Figure S2. SNP matrix obtained from core-genome DNA comparison between *K. pneumoniae* ST11 KL105 genomes supporting phylogenetic tree.
